# Confirmation of Bioinformatics Predictions of the Structural Domains in Honeybee Silk

**DOI:** 10.3390/polym10070776

**Published:** 2018-07-16

**Authors:** Andrea L. Woodhead, Andrew T. Church, Trevor D. Rapson, Holly E. Trueman, Jeffrey S. Church, Tara D. Sutherland

**Affiliations:** 1 CSIRO Manufacturing, Pigdons Rd, Waurn Ponds, VIC 3216, Australia; Jeff.church@jpascientific.com; 2 JPA Scientific, P.O. Box 2573, Chino Hills, CA 91709, USA; Andrew.church@jpascientific.com; 3 CSIRO Health and Biosecurity, Clunies Ross St, Black Mountain, ACT 2601, Australia; Trevor.rapson@csiro.au (T.D.R.); Trueman.holly@gmail.com (H.E.T.); Tara.sutherland@csiro.au (T.D.S.)

**Keywords:** coiled coil, protein secondary structure, bioinformatics protein folding prediction, protein design, protein engineering, protein materials, infrared spectroscopy, circular dichroism spectroscopy, molecular dynamics, cast film solubility

## Abstract

Honeybee larvae produce a silk made up of proteins in predominantly a coiled coil molecular structure. These proteins can be produced in recombinant systems, making them desirable templates for the design of advanced materials. However, the atomic level structure of these proteins is proving difficult to determine: firstly, because coiled coils are difficult to crystalize; and secondly, fibrous proteins crystalize as fibres rather than as discrete protein units. In this study, we synthesised peptides from the central structural domain, as well as the N- and C-terminal domains, of the honeybee silk. We used circular dichroism spectroscopy, infrared spectroscopy, and molecular dynamics to investigate the folding behaviour of the central domain peptides. We found that they folded as predicted by bioinformatics analysis, giving the protein engineer confidence in bioinformatics predictions to guide the design of new functionality into these protein templates. These results, along with the infrared structural analysis of the N- and C-terminal domain peptides and the comparison of peptide film properties with those of the full-length AmelF3 protein, provided significant insight into the structural elements required for honeybee silk protein to form into stable materials.

## 1. Introduction

Honeybee larvae spin silken cocoons in which they pupate. These cocoons accumulate in the honeybee hive and form the basis of the brood comb for successive generations of larvae. The accumulating silk contributes to the thermal and mechanical stability of the hive [1].

Honeybee silk has a distinctive molecular structure that is unlike the primarily β-sheet structure of spider and silkworm silks. Fibres drawn from honeybee silk glands are predominantly coiled coil with much lower levels of β-sheet conformation (summarised in Sutherland et al. [2]). In contrast, native honeybee silk remaining in the presence of wax has a molecular structure consisting of isolated α-helices rather than coiled coils [3].

Native honeybee silk is composed of four fibrous silk proteins, all similar in length, architecture, and amino acid composition [4]. Homologs of all four proteins have been found in all aculeate (bee, ant, hornet) species investigated to date from the superfamilies Apoidea and Vespoidea [5]. The honeybee silk proteins are small (~30 kDa) in comparison with silkworm and spider silk proteins (~250–400 kDa), with relatively low levels of primary sequence repetition, and hence full-length silk proteins are readily expressed in recombinant systems [6,7,8]. Recombinant versions of one of the honeybee silk proteins, AmelF3 (*Apis mellifera* Fibrion 3), self-assembles into coiled coil units that can be fabricated into a variety of material forms [9,10,11,12], making it of interest for the design of functionally active protein-based materials [13,14,15].

Rational design of functional materials requires an understanding of the atomic structure of the protein template. We have not been able to generate atomic level structural data for these proteins, with the protein crystallising as fibres rather than as individual protein units. With the rapid advances of *in silico* techniques, bioinformatics structural prediction programs are becoming commonly used tools for developing models of protein structure. The prediction program MARCOIL [16] confidently predicts the position of the central 216 residues (out of a total of 315 residues) of the AmelF3 honeybee silk protein within a coiled coil structure, however, other coiled coil prediction programs such as Coils [17], Paircoil [18], and Multicoil [19] are less confident (see Supplementary Information Figure S1).

The sequences of the coiled coil silk proteins are atypical in comparison with canonical coiled coil proteins [20]. In particular, they have a high frequency of alanine, rather than more hydrophobic residues such as leucine and isoleucine, in the predicted coiled coil core positions. It is likely that this anomaly prevents confident prediction in programs other than MARCOIL, as these prediction programs are based on sequence comparisons with known coiled coils, as proposed by Parry in 1982 [21]. In contrast, MARCOIL utilizes a hidden Markov model that exploits the information contained in multiple sequence alignments to predict coiled-coil regions. It is also noted that Coils and Paircoil are used to predict dimeric coiled coils, and Multicoil, is used to predict trimeric coiled coils, whereas the native honeybee silk is a tetrameric coiled coil [22,23].

In homologous hornet silk proteins, the central region of the proteins are alanine-rich, whereas the N- and C-terminal domains are serine-rich. Solid-state NMR spectroscopy has found that the alanine-rich regions of the proteins are associated with α-helical structure, whereas the serine-rich N- and C-terminal domains are predominately β-sheet in structure [24,25,26,27]. In AmelF3, there is no such distinction between the central and terminal ends. To confirm bioinformatics coiled coil predictions, we compared the structural behaviour, as determined by circular dichroism spectroscopy, infrared spectroscopy, and molecular dynamics, of peptides from the central region of AmelF3. These results, along with the infrared structural analysis of the N- and C-terminal domain peptides and the comparison of peptide film properties to those of the full-length AmelF3 protein, provided significant insight into the structural elements required for honeybee silk protein to form into stable materials.

## 2. Materials and Methods 

### 2.1. Samples

**Figure 1:**
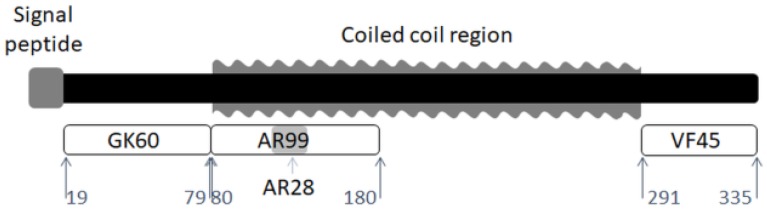
Schematic representation of the locations of the peptides used in this study within the honeybee silk protein AmelF3.

We obtained peptides from Mimotopes (Clayton, Australia) that corresponded to the predicted structural regions in the honeybee silk protein (AmelF3; NCBI accession no: NP_001129680), as shown in Figure 1. The peptides were provided as lyophilised powders that can be readily re-suspended in solution at the required concentrations. AR99 corresponds to the first 14 heptads of the predicted coiled coil region (A80–R180 of the full length protein; AAAVVKA SALALAEA TVAQNVA SDLQKR); AR28 corresponds to the four central heptads of AR99 (AQQAQ LNAQEKSL AALKAQSEE EAASAR); GK60 corresponds to the N-terminal end of the protein; and VF45 corresponds to the C-terminal end of the protein. Full length AmelF3 honeybee silk protein was prepared as described in Weisman et al. [8] and dialysed against water overnight at 4 °C to remove sodium dodecyl sulfate.

### 2.2. Fabrication Methods 

Peptides or protein, as 1% (weight/volume) solutions in water, were cast onto plastic trays and allowed to dry at room temperature overnight, generating transparent films. The cast films were treated with aqueous 70% methanol, a treatment used to stabilise artificial honeybee silk materials [9].

### 2.3. Fourier Transform Infrared (FTIR) Spectroscopy

Fourier Transform Infrared (FTIR) spectra from films cast from peptide solutions were obtained at 4 cm^−1^ resolution using a Perkin Elmer System 2000 FTIR fitted with a ZnSe single bounce attenuated total reflectance accessory (MIRacle ATR, Pike Technologies, Fitchburg, WI, USA). A mercury cadmium telluride liquid nitrogen cooled detector was used. A minimum of 32 scans were co-averaged. Spectra obtained from four areas of each cast film were found to be highly reproducible.

Spectral manipulation was carried out using GRAMS AI v 9.1 (Thermo Fisher Scientific Inc., Waltham, MA, USA) as described in Weisman et al. [8] for honeybee silk. Spectra were baseline corrected by applying a linear offset at 1775 cm^−1^ and then were normalized on the strongest amide I feature. This manipulation also served to normalise the C–H modes, the intensities of which are not expected to be affected by conformational changes. Amide I band components were identified by both second derivative spectroscopy and Fourier self-deconvolution methods. Fits for similar amide I band contours, for example, the methanol–water treated peptides, were initiated from the same base set of band components.

### 2.4. Circular Dichroism (CD) Spectroscopy

Far-UV circular dichroism (CD) spectra were collected from peptides at various concentrations in water with 10 mM NaCl (pH 7.2) using a 0.2 mm path-length cell (20/O/Q/0.2, Starna, Atascadero, CA, USA) in a Chirascan spectrometer (Applied Photophysics, Leatherhead, UK) at room temperature. Spectra were collected between 190–250 nm with 1 nm increments and an integration time of 0.5 s.

### 2.5. Molecular Dynamics (MD) Simulations

All molecular dynamics (MD) simulations were carried out in the Gromacs 5.1.4 molecular dynamics simulation package [28]. The peptide and ion forces were represented using the CHARMM 22* forcefield [29], with water molecules represented via the TIPs3P water model [30]. For all MD simulations, Newton’s equations of motion utilized a 1 fs time step integrated via the leapfrog algorithm [31], with data from the production runs recorded every 10,000 stimulation steps. All systems were modeled at a temperature of 300 K, maintained using the Nosé–Hoove thermostat [32,33,34] with a coupling constant of 0.2 ps, while a pressure of 1 bar was maintained via the Parrinello–Rahman barostat with a coupling constant of 0.4 ps [35]. Electrostatics were summed via the particle mesh Ewald method [36], with Lennard Jones forces represented via a force-switch with a cut-off starting at a distance of 1.0 nm and ending at a distance of 1.2 nm.

All systems were modeled in a cubic box with side dimensions of ~9.0 nm with periodic boundary conditions in all directions. Each system contained 24,700 water molecules and a number of Na^+^ ions equal to the number of peptides in the system to counter balance the peptide charge. The initial conformation for the individual peptides modeled encompassed either an α-helical conformation, wherein each of the peptides back bone phi (Φ) and psi (Ψ) dihedral angles were set to −60° and −45°, respectively, or an extended conformation wherein each of the peptides phi and psi backbone dihedral angles was set to 180° and −180°, respectively, with the exception of the dihedral angles of Ala14 and Ala15, which together formed a beta turn via phi and psi dihedral angles of 80° and 0°, respectively.

Once systems containing the peptides, water, and ions were generated, an initial geometry optimization was carried out on each system via the steep integrator with completion after either 1,000,000 optimization steps or when an energy tolerance of 1000 kJ was achieved. After each system was geometry optimized, a 50 ps run was carried out in the NVT ensemble to allow the system to relax to the target temperature. Following this, each system was brought to the target pressure via 100 ps NPT run, the final frames from which were utilized as starting configurations for the production runs.

All images obtained via computational study depicted in this work share the following color scheme unless otherwise specified. The AR28 N-terminal alanine is shown in orange; the peptide backbone atoms are depicted as the cyan tube; Ser12, Ser20, and Ser26 sidechains are colored magenta; and all other sidechains and their respective α-carbons are shown in black.

## 3. Results

### 3.1. Structure of Peptides in the Solid-State

**Figure 2:**
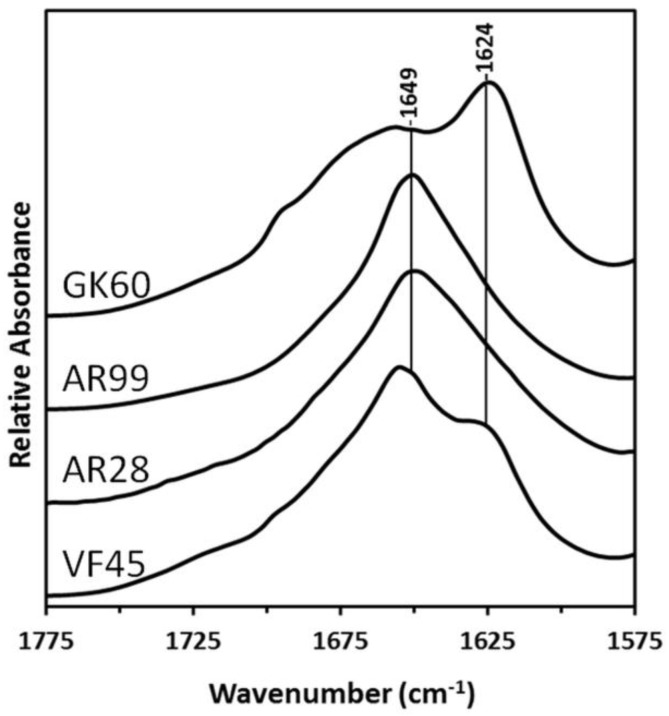
Amide I regions of the FTIR spectra obtained from cast films of the four peptides derived from different regions of the honeybee silk protein, as shown in Figure 1. The dominant coiled coil (1649 cm^−1^) and β-sheet (1624 cm^−1^) bands are indicated. Deconvolution peaks are shown in Supplementary Information Figure S2 and the proportion of each structure is shown in Table 1.

FTIR spectroscopy was utilized to determine the secondary structure of peptides derived from different regions of the honeybee silk protein AmelF3 (Figure 1), in the solid-state. While the correlation of amide I and III band components and α-helical, β-sheet, β-turn, and random coil secondary structures have been extensively studied [37,38], the coiled coil structure has been the subject of only a few papers [8,39], demonstrating that it can be distinguished from the less complex α-helical structure. The amide I regions of the spectra obtained from cast films of the four peptides are shown in Figure 2 and their deconvolutions are shown in Supplementary Information Figure S2. A summary of the proportions of the secondary structures present in the peptides is presented in Table 1.

Analysis of the FTIR spectra indicated that the two peptides from the honeybee silk protein’s predicted coiled coil region did indeed form significant levels of coiled coil molecular structures in the “as cast” solid-state (AR99 and AR28; Table 1). This is most evident by the dominant 1649 cm^−1^ amide I band maxima [8,39] observed from these peptides (Figure 2). In the spectra obtained from the terminal domains (GK60 and VF45; Figure 2), β-sheet conformation is more apparent with strong, well defined features near 1624 cm^−1^ [37,38].

Naturally occurring honeybee silk contains some β-sheet structure, which is believed to cross-link the coiled coils, thereby stabilising the material form [20]. β-sheet structures can be generated in artificial materials through aqueous methanol [9] or water annealing [12] treatments, which result in the material becoming insoluble in aqueous solvents. The effect of aqueous methanol stabilization treatment on the peptide molecular structure was also assessed by FTIR spectroscopy (Table 1 and Supplementary Information Figure S1). Aqueous methanol treatment lead to small relative increases in the amount of β-sheet structures in the peptides from the N- and C-terminal regions with corresponding loss of other structures. In the peptides from the central region, aqueous methanol treatment destabilised the coiled coil structure so that it was reduced from over 80% to less than 20% of the structural components.

### 3.2. Structure of Peptides in Solution

**Figure 3:**
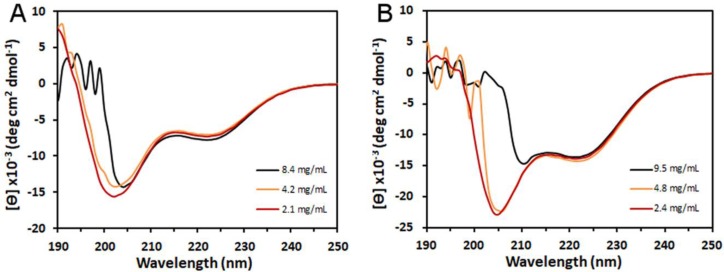
Circular dichroism spectra for peptides AR28 (**A**) and AR99 (**B**) as a function of concentration.

CD spectroscopy is commonly used to yield information about proteins in the solution state [40,41]. At concentrations below 5 mg/mL, the CD spectra from the peptides had maxima below 200 nm and minima around 203 and 220 nm, suggesting a randomly coiled structure (Figure 3). As the concentrations increased, the maxima below 200 nm was lost and the 203 nm minimum shifted to 208 nm, becoming significantly more negative than the 220 minima, consistent with the peptides forming a helical structure. The flattening of the CD signal below 210 nm is a symptom that the samples are becoming too concentrated and analysis of more concentrated samples was not meaningful. The profiles of these spectra are consistent with spectra previously obtained from lower concentrations of the European honeybee silk protein, AmelF3 [40], and the Asian honeybee silk proteins, Ador1–4 [7].

**Figure 4:**
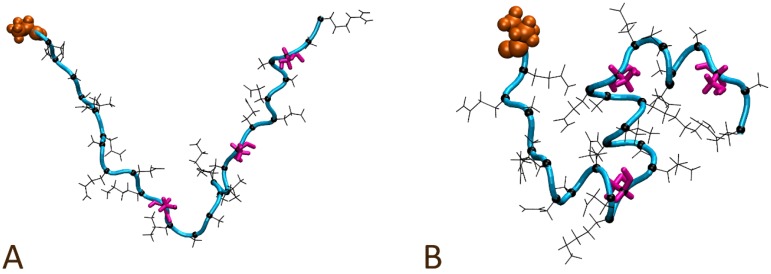
A solution bound single AR28 peptide modeled in an initially extended conformation (**A**) and the final observed conformation (**B**) after 100 ns. The N-terminal alanine is shown in orange; the peptide backbone atoms are depicted as the cyan tube; Ser12, Ser20, and Ser26 sidechains are colored magenta; and all other sidechains and their respective α-carbons are shown in black.

Single AR28 peptides were modeled to gain insight into the potential conformational preferences of the peptide in solution. Both unfolded and α-helical initial conformations were investigated via 100 ns MD simulations in the NVT ensemble. The first system modeled was a single AR28 peptide in an initially extended conformation, as seen in Figure 4A. While the initial conformation lacked any apparent α-helical turns, after a total simulation time of 100 ns, visual inspection clearly suggests the formation of three regions containing complete α-helical turns, which can be seen in Figure 4B and are described in Table 2. Other regions of the peptide may have briefly adopted α-helical turns over the course of the simulation, but these appeared to have been minimal and largely transient.

Helical turns in region 1 appeared to initially form starting after a total simulation period of ~35 ns, however, they appeared to rapidly unfold ~15 ns later. Region 2 appeared to take on α-helical secondary structure after a total of 50 simulated ns and remained present until the completion of the simulation. Region 3 appeared to adopt α-helical turns shortly after region 2, starting after a total of 55 simulated ns and remaining until the completion of the simulation. Finally, region 1 appeared to readopt α-helical turns after a total of 75 simulated ns, which then remained until the completion of the simulation. It is possible that the stability of α-helical content in region 1 is in part controlled via the formation of helical turns in regions 2 and 3.

Quite striking is how each of the three regions is separated by single serine residues (Ser12, Ser20, and Ser26) that did not appear to readily adopt the α-helical secondary structure. This appears to have prevented the formation of a single long α-helical coil spanning the entire peptide. While it was not observed during the course of this simulation, the possibility of the serine residues adopting the α-helical secondary structure as part of a larger helix is not ruled out. To investigate this possibility, a single AR28 peptide in solution with an initially α-helical conformation was modeled, as seen in Supplementary Information Figure S3A.

The initial conformation for this simulation encompassed a complete helix across the entire peptide sequence. The peptide’s final conformation after 100 ns was largely α-helical across the entire length of the sequence, including the sequence’s serine residues as shown in Supplementary Information Figure S3B. Over the course of 100 ns, the AR28 peptide did not display any significant unfolding, largely remaining α-helical across the entire sequence. Together with the previous observations, it could be suggested that α-helical folding of the serine residues presents significant thermodynamic barriers on the AR28 energy landscape that the sequence must overcome to reach a fully α-helical conformation. However, it would likely require additional study via the use of advance sampling techniques to confirm if a completely α-helical conformation is indeed representative of the AR28 native state.

**Figure 5:**
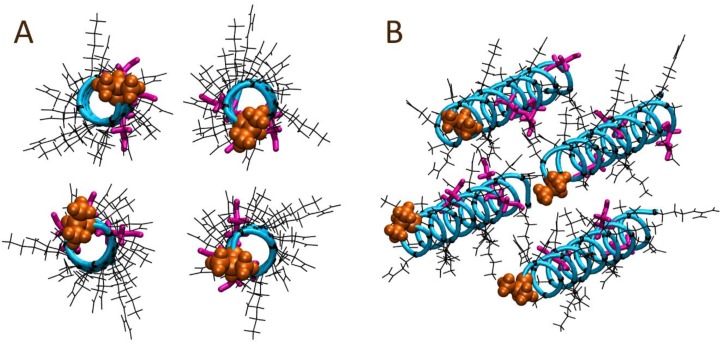
Starting peptide alignment for the solution bound four AR28 peptide system modelled with a head-to-head alignment: (**A**) front and (**B**) perspective views. The N-terminal alanine is shown in orange; the peptide backbone atoms are depicted as the cyan tube; Ser12, Ser20, and Ser26 sidechains are colored magenta; and all other sidechains and their respective α-carbons are shown in black.

To gain insight into the coming together of four solution bound AR28 peptides into a coiled coil quaternary structure, 50 ns MD simulations in the NVT ensemble were carried out. These systems utilized four copies of the AR28 peptide, each of which was initially in a completely α-helical conformation. The system started with each of the four peptides aligned roughly in parallel with all N-terminals together, and all C-terminals together as depicted in Figure 5, and hence forth is referred to as a head-to-head alignment.

**Figure 6:**
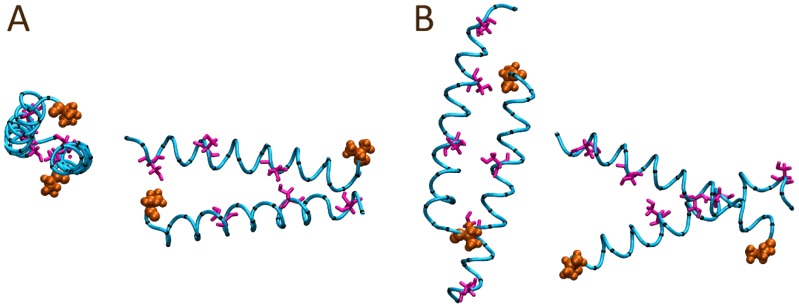
Final peptide alignment of the solution bound four AR28 peptide system modelled with an initial head-to-head alignment after 100 ns: (**A**) front and (**B**) side views. The N-terminal alanine is shown in orange; the peptide backbone atoms are depicted as the cyan tube; Ser12, Ser20, and Ser26 sidechains are colored magenta; and all other sidechain α-carbons are shown in black with all other sidechain atoms hidden for ease of viewing.

While initially starting in a square, box-like conformation with all N-terminals together and all C-terminals together, within the first ns of simulation time, the peptides appeared to quickly separate starting from the N-terminal, as well as by a spatial translation of the peptides along their lengths. Beyond this point in the trajectory, the peptides appeared to drift away from each other sufficiently such that the sequences could re-orientate. By the end of 50 ns, the four peptides had split into two pairs, both of which appeared to encompass a head-to-tail orientation, as shown in Figure 6.

**Figure 7:**
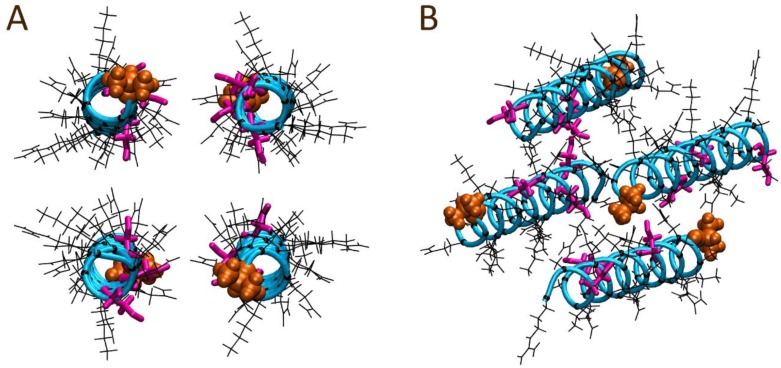
Starting peptide alignment for the solution bound four AR28 peptide system modelled with a head-to-tail alignment: (**A**) front and (**B**) perspective views. The N-terminal alanine is shown in orange; the peptide backbone atoms are depicted as the cyan tube; Ser12, Ser20, and Ser26 sidechains are colored magenta; and all other sidechains and their respective α-carbons are shown in black.

**Figure 8:**
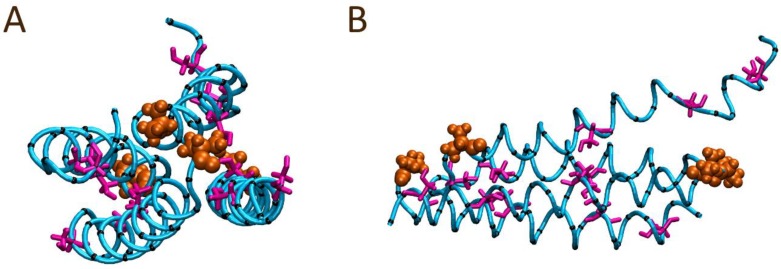
Final peptide alignment of the solution bound four AR28 peptide system modelled with an initial head-to-tail alignment after 100 ns: (**A**) front and (**B**) side views. The N-terminal alanine is shown in orange; the peptide backbone atoms are depicted as the cyan tube; Ser12, Ser20, and Ser26 sidechains are colored magenta; and all other sidechain α-carbons are shown in black with all other sidechain atoms hidden for ease of viewing.

To contrast, a second four peptide system was constructed utilizing a similar parallel arrangement; however, the orientations of two of the peptides were flipped to form a head-to-tail arrangement as depicted in Figure 7. Within the first ns of the simulation, minor shifts of the peptides towards each other were observed, as well as minor spatial shifting along the peptides’ lengths. Over the course of 50 ns, the initially parallel conformation was largely maintained with no significant separation of the peptides observed. Visual inspection at the end of the 50 ns trajectory (Figure 8) suggested minor twisting of the peptides around each other, forming what looks like the basis of a coiled coil arrangement. To quantify the formation of coiled coil structure within the final frame of the simulation trajectories, the SOCKET algorithm has been utilized [42]. The analysis identified a four-strand antiparallel coiled coil region for the head-to-tail system configuration, while only a single antiparallel two-strand coiled coil region was identified for the head-to-head system configuration. SOCKET analysis of the initial frames of the trajectories did not detect coiled coil structure.

The results of the simulation work suggest that the AR28 peptides form a coiled coil structure in solution via a head-to-tail alignment. Whether this is due to alignments of the different side chains, the formation of a hydrophobic core, the attraction and repulsion of termini charges, or a combination of all of these factors is not clear from the current study.

It is plausible that longer segments of the coiled coil region, such as AR99, may result in larger numbers of side chain interactions or the formation of a larger hydrophobic core that may make it thermodynamically favorable for the peptides to align in a head-to-head arrangement.

While the SOCKET analysis identified a four-strand antiparallel coiled coil region for the head-to-tail AR28 system configuration, we note that the high alanine content of the cores does not require knobs-into-holes interactions [20]. High levels of core alanines, by virtue of their small side chains in comparison with canonical core-occupying residues, can alter the nature of core layer packing. The small side chains enable greater packing flexibility, allowing some otherwise disallowed coiled coil structures. Theoretical models [43] and examination of crystal structures [43,44,45] indicate a deviation from the knobs-into-holes pattern for alanine. The smaller alanine side-chain can be completely accommodated within one half of the usual hole, preferentially packing within the smaller triangular hole defined by three residues on the opposing helix. Fulfilling this preference requires the incorporation of an axial stagger; a shift of one helix along the super-helical axis relative to the other. Because of this atypical core composition, we believe that SOCKET may not be the best way to analyze these structures.

### 3.3. The Role of the N- and C-Terminal Regions in Material Formation

**Figure 9:**
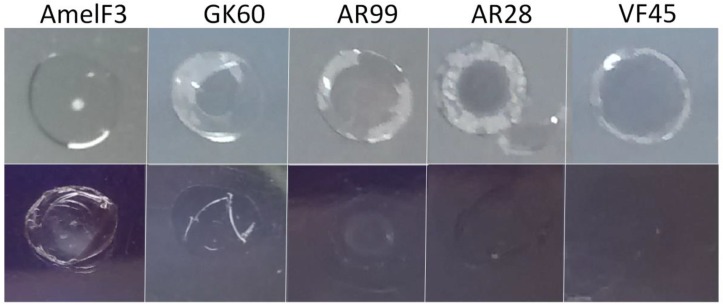
Images of films cast from full-length honeybee silk protein (AmelF3), and peptides from different regions (Figure 1). **Top panel**, as cast films; **Bottom panel**, after aqueous methanol treatment, where materials cast from peptides have dissolved and are no longer obvious.

In order to determine if the N- and C-terminal regions were required to generate stable solid-state materials, films were cast from full-length protein, as well as from the various peptides (Figure 9). The full-length protein produced a transparent, flexible film as previously described [11]. Similarly, the film cast from the longer peptide from the central region (AR99) formed a transparent, flexible film. Films cast from the other peptides were brittle, showed disfiguration around the edges, and could not be removed from the plastic plate without breaking (Figure 9 top panel).

The cast films were treated with aqueous methanol, using the method developed to stabilise material forms generated from full-length honeybee silk protein [9]. Whereas aqueous methanol treatment stabilised the film cast from the full-length protein solution, making it water insoluble, similar treatment of the films produced from the peptides resulted in their dissolution (Figure 9 bottom panel).

## 4. Discussion

Determining the atomic structure of fibrous proteins, such as silks, presents a unique challenge [46]. While bioinformatics tools can provide useful insights through structural predictions, it is important to validate these predictions experimentally. Our results confirm a central coiled coil region in AmelF3, which is in agreement with predictions from the prediction program MARCOIL [16].

In addition, our results highlight two important characteristics of the honeybee silk proteins. The first is the importance of the N- and C-terminal regions in forming a stable solid-state material. In full-length AmelF3, as has been demonstrated in homologous proteins, we presume that the β-sheets form at the terminal ends of the protein with the central domain maintaining a coiled coil structure.

A second point of interest is the relationship between the length of the coiled coil forming peptides and the concentrations required to form the coiled coil structure. The sequences of numerous silk proteins homologous to the honeybee AmelF3 protein are known [5]. While the sequences of these silk proteins have diverged considerably, the length of the predicted coiled coil region has been highly conserved [5], implying that it has been functionally optimized. Formation of the coiled coil structure in full-length honeybee silk proteins occurs at around 4 mg/mL [40]; concentrations significantly higher than what is required for classical leucine zipper coiled coils [47]. Even higher concentrations are required before the coiled coil is observed in the peptides, with the coiled coil structure detected by FTIR after the peptides have been concentrated when cast as films.

The high protein concentrations required before coiled coils are achieved with these peptides highlights the importance of the length of the coiled coil region for protein folding and stability. Increased length is associated with increased coiled coil stability. However, there are other ways to increase stability such as increasing hydrophobicity of the core residues or incorporating complimentary charged residues in the positions adjacent to the core, neither of which are apparent in the silk proteins. For example, the silk coiled coil domain has predominantly alanine and serine in the core positions [8], whereas leucine zippers have predominantly leucine and isoleucine [47].

We propose that the length of the coiled coil domain has evolved as to be optimal, in relation to the width of the coiled coil unit, for native materials fabrication. The model for fabrication of the coiled coil silks by insects is that highly anisotropic tetrameric coiled coils in the silk gland direct local molecular alignment prior to silk production (Walker et al., 2015) [48]. This model is supported by several studies that have shown local alignment of the protein units in the glands of bees and ants [2,49]. The coiled coil region of the silk proteins is absent of stutters, stammers, or other structural distortions, implying formation of an undistorted rod-like structure [50], consistent with the ability of the protein units to pack closely.

Upon extrusion of the silk protein dope from the silk gland, dehydration allows electrostatic interactions (and sometimes covalent bonding; Campbell et al. [5]) between the coiled coil units, which are then aligned in the direction of flow through the spinneret to produce fibres where the coiled coils are aligned in the direction of the fibre. In this model, a key function of the coiled coil structure is to provide local alignment of the protein units prior to material fabrication, and it is likely that the length-to-width ratio of the coiled coil domain has been optimised to facilitate the organisation and flow of structures in the silk gland. The role of the coiled coil molecular structure in fabrication is supported by the finding that the coiled coil is not present in the final wax/silk composite material used in the honeybee hive [3].

According to this model the folding behaviour of the proteins would be required to have evolved to match the silk gland environment and fabrication process. The native coiled coil unit in honeybee silk is a tetrameric coiled coil [22], most likely a hetrotetrameric coiled coil [23]. In the environment of the silk gland, which contains very high levels of silk protein, folding at relatively high concentrations may facilitate correct folding of the four different proteins into the correct structure for subsequent mesophase ordering of the coiled coil units.

## 5. Conclusions

Experimental data obtained from the central peptides derived from honeybee silk protein were consistent with bioinformatics predictions of protein structure, giving confidence to protein engineers interested in using this protein for the design of new materials. In solution, CD data suggests that the peptide corresponding to four predicted heptads folds into a helical structure at around 8 mg/mL, and the peptide containing ten predicted heptads folds into a helical structure at around 9 mg/mL. These are significantly higher concentrations compared with that for the full protein, which adopts a coiled coil structure at 4 mg/mL. This folding behaviour can be correlated to the length of the peptide, demonstrating the importance of the length of the coiled coil domain on coiled coil stability.

Unlike that of materials formed from the full-length proteins, materials generated from the peptides were not stable in water, suggesting that both the coiled coil regions of the protein and the N- and C-terminal regions of the protein are required to form a stable material. This finding is consistent with the N- and C-terminal domains generating β-sheet structures to cross-link the coiled coil structures into a stable material format.

## Figures and Tables

**Table 1:** Molecular structure of peptides from different regions of the honeybee silk protein, determined from deconvolution (Supplementary Figure S2) of the Fourier Transform Infrared (FTIR) spectra of cast films (Figure 2) before and after methanol treatment.

Conformation (%)	GK60 (N-Terminal)		AR99 (Coiled Coil)		AR28 (Coiled Coil)		VF45 (C-Terminal)	
	CF	MeOH	CF	MeOH	CF	MeOH	CF	MeOH
Coiled coil	14	12	86	18	85	13	19	10
β-sheet	50	59	0	49	0	54	44	65
β-turn	28	23	0	23	0	28	28	19
Unordered	9	6	14	9	15	5	10	6

CF: “as cast” film; MeOH: after 70% methanol treatment.

**Table 2:** Descriptions detailing the three α-helix supporting regions observed for the solution bound single AR28 peptide modeled from an initially extended conformation, as shown in Figure 4.

α-Helix Region	Starting Residue	Ending Residue
1	Leu6	Lys11
2	Leu13	Gln19
3	Glu21	Ala25

## Supplementary Materials

The following are available online at http://www.mdpi.com/2073-4360/10/7/776/s1, Figure S1. Bioinformatics predictions for the honeybee silk protein AmelF3: (**A**) MARCOIL and (**B**) Paircoil, Figure S2. Deconvolution of the amide I regions of the infrared spectra obtained from “as cast” (**top**) and 70% methanol treated (**bottom**) films of the peptides: (**A**) GK60, (**B**) AR99, (**C**) AR28, and (**D**) VF45, Figure S3. A solution bound single AR28 peptide modeled in an initially α-helical conformation (**A**) and the final observed conformation (**B**) after 100 ns.
