# Supplementary material for: Confirmation of Bioinformatics Predictions of the Structural Domains in Honeybee Silk
Source: Polymers (Basel). 2018 Jul 16;10(7):776. doi: 10.3390/polym10070776 (PMC6403662; doi:10.3390/polym10070776)
Supplement: Supplementary file 1 [file polymers-10-00776-s001.pdf]

Confirmation of Bioinformatics Predictions of the Structural Domains in Honeybee Silk

Andrea L. Woodhead [1,*], Andrew T. Church [2], Trevor D. Rapson [3], Holly E. Trueman [3], Jeffrey S. Church [1,2] and Tara D. Sutherland [3]

1 CSIRO Manufacturing, Pigdons Rd, Waurn Ponds, VIC 3216, Australia; Jeff.church@jpascientific.com (J.S.C)
2 JPA Scientific, P. O. Box 2573, Chino Hills, CA 91709, USA; Andrew.church@jpascientific.com (A.T.C.)
3 CSIRO Health and Biosecurity, Clunies Ross St, Black Mountain, ACT 2601, Australia; Trevor.rapson@csiro.au (T.D.R.); Trueman.holly@gmail.com (H.E.T.); Tara.sutherland@csiro.au (T.D.S.)
* Correspondence: Andrea.Woodhead@csiro.au (A.L.W.); Tel.: +61-(3)-5246-4766

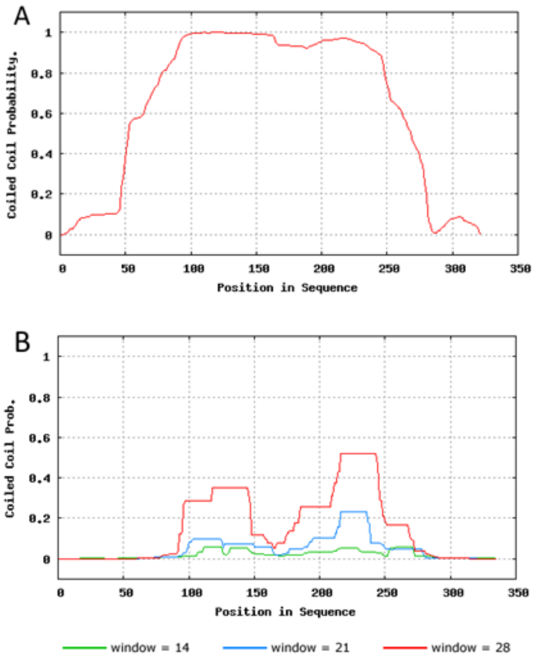

Figure S1. Bioinformatics predictions for the honeybee silk protein AmelF3: A) MARCOIL and B) Paircoil.

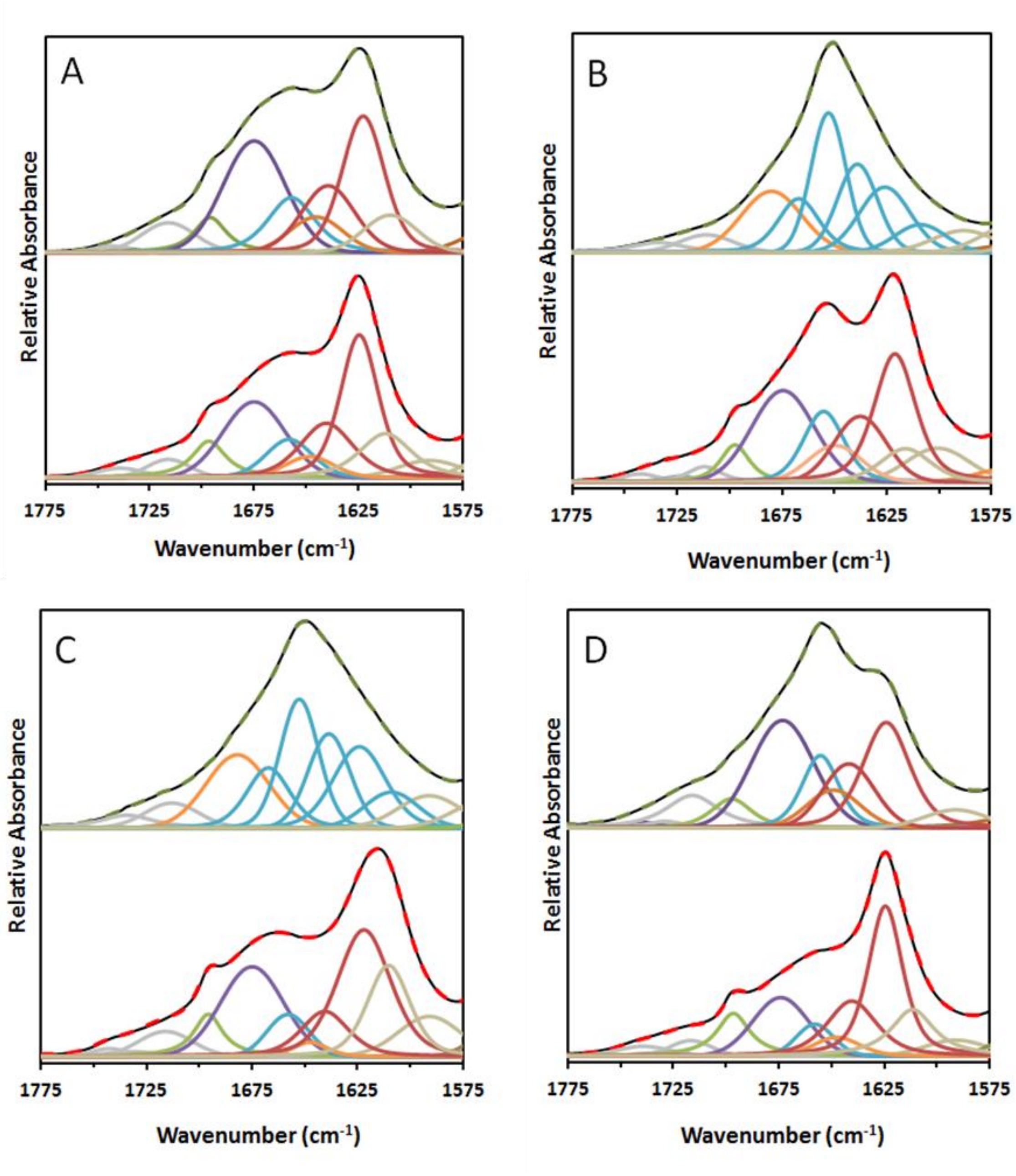

Figure S2. Deconvolution of the amide I regions of the infrared spectra obtained from "as cast" (top) and 70% methanol treated (bottom) films of the peptides: A) GK60, B) AR99, C) AR28, and D) VF45. The top solid black lines are the raw spectra with constructed spectra shown as green (as cast films) or red (methanol treated films) dotted spectra (in all cases, this overlies the raw spectra). Coiled coil bands are shown in blue, β-sheet bands are shown in brown, β-turn bands in purple, antiparallel β-sheet bands in dark green, unordered structural bands in orange, and carbonyl bands in grey.

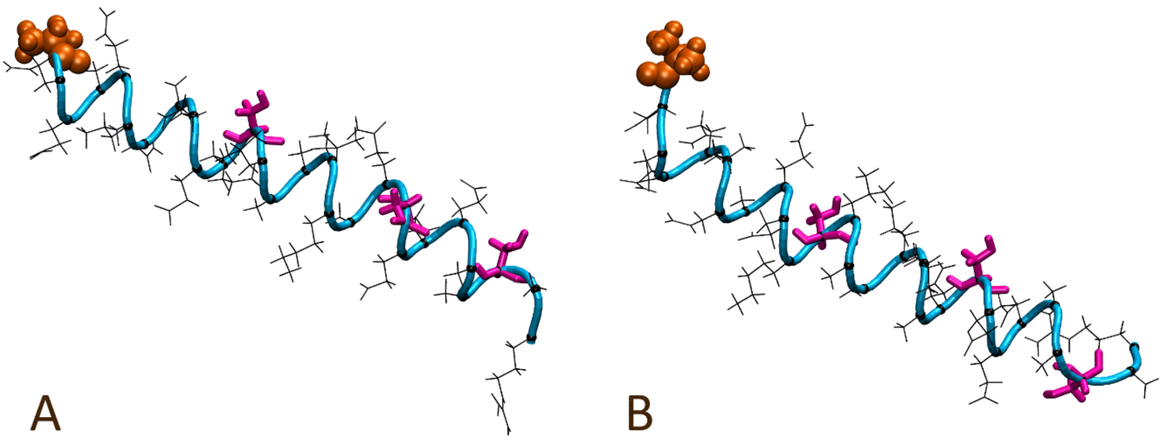

Figure S3. A solution bound single AR28 peptide modeled in an initially α-helical conformation (A) and the final observed conformation (B) after 100 ns.
